# Optimizing Strontium Ruthenate Thin Films for Near-Infrared Plasmonic Applications

**DOI:** 10.1038/srep09118

**Published:** 2015-03-13

**Authors:** Laurentiu Braic, Nikolaos Vasilantonakis, Bin Zou, Stefan A. Maier, Neil McN. Alford, Anatoly V. Zayats, Peter K. Petrov

**Affiliations:** 1 Department of Materials, Imperial College London, Prince Consort Road, London SW7 2AZ, UK; 2 Department of Physics, King's College London, Strand, London WC2R 2LS, UK; 3 Department of Physics, Imperial College London, Prince Consort Road, London SW7 2BB, UK

## Abstract

Several new plasmonic materials have recently been introduced in order to achieve better temperature stability than conventional plasmonic metals and control field localization with a choice of plasma frequencies in a wide spectral range. Here, epitaxial SrRuO_3_ thin films with low surface roughness fabricated by pulsed laser deposition are studied. The influence of the oxygen deposition pressure (20–300 mTorr) on the charge carrier dynamics and optical constants of the thin films in the near-infrared spectral range is elucidated. It is demonstrated that SrRuO_3_ thin films exhibit plasmonic behavior of the thin films in the near-infrared spectral range with the plasma frequency in 3.16–3.86 eV range and epsilon-near-zero wavelength in 1.11–1.47 μm range that could be controlled by the deposition conditions. The possible applications of these films range from the heat-generating nanostructures in the near-infrared spectral range, to metamaterial-based ideal absorbers and epsilon-near-zero components, where the interplay between real and imaginary parts of the permittivity in a given spectral range is needed for optimizing the spectral performance.

Plasmonic nanostructures and metamaterials open up unprecedented possibilities to control and manipulate light at the nanoscale^1^. Plasmonic phenomena originate from the collective oscillations of free electrons interacting with electromagnetic fields near the surface and require materials with high concentration of free electrons and low loss, such as Ag and Au metals. Modern technological requirements on the physical and chemical properties of plasmonic materials, such as high temperature stability, compatibility with CMOS semiconductor processing to name but a few, have stimulated a search for alternative plasmonic media^2^,^3^. Plasmonic properties of ITO and doped-ZnO conductive oxides and TiN nitrides have already been demonstrated^4^,^5^. At the same time, new applications were put on the agenda, such as perfect absorbers and the so-called epsilon-near-zero (ENZ) effects where the interplay between real and imaginary parts of permittivity is essential for flexibility of the design and achieving required light penetration in the material needed for heat and hot-electron generation.

SrRuO_3_ (SRO), a material with perovskite-type crystal structure, has been the subject to intense research due to its high thermal and electrical conductivity, and high thermal and chemical stability (up to 1200 K in oxidizing or inert-gas atmospheres)^6^. For these reasons SrRuO_3_ is widely used as electrode material for ferroelectric devices and semiconductors^7^,^8^. and can be grown on, or used as a standard substrate for the epitaxial growth of, SrTiO_3_ and other functional materials^9^,^10^,^11^,^12^; it can also be grown on Si substrates^13^. Additionally, it is used in heterostructure electronic devices as buffer layer for deposition of high-temperature superconductors and ferroelectric materials^8^,^14^.

SrRuO_3_ exhibits carrier dynamic similar to that of the cuprate superconductors^15^,^16^,^17^. The mid-infrared optical conductivity is a common feature of all ruthenium oxides, including SrRuO_3_^18^,^19^,^20^,^21^. In general, the experimental results for itinerant electron systems on the kinetic energy of the electrons, as derived from optical conductivity data are in good agreement with band-structure calculations with the deviations due to strong spin-orbit coupling, which are specific for many intermetallic compounds with d and f electrons^22^.

Considering the above described electronic properties, this article examines the potential of SrRuO_3_ thin films for plasmonic applications in the near-infrared spectral range, including optical telecommunication frequencies. We show that the optical properties of the films can be controlled via growth conditions changing oxygen pressure during film deposition, and the epsilon-near-zero condition can be controllably achieved in the wavelength range 1.11–1.47 μm, so that a negative permittivity and plasmonic behavior are observed at longer wavelengths. The balance of plasmonic properties and loss in the telecom wavelength range makes this material competitive for design of ENZ and perfect-absorber-type metamaterial applications.

## Results

**Figure 1:**
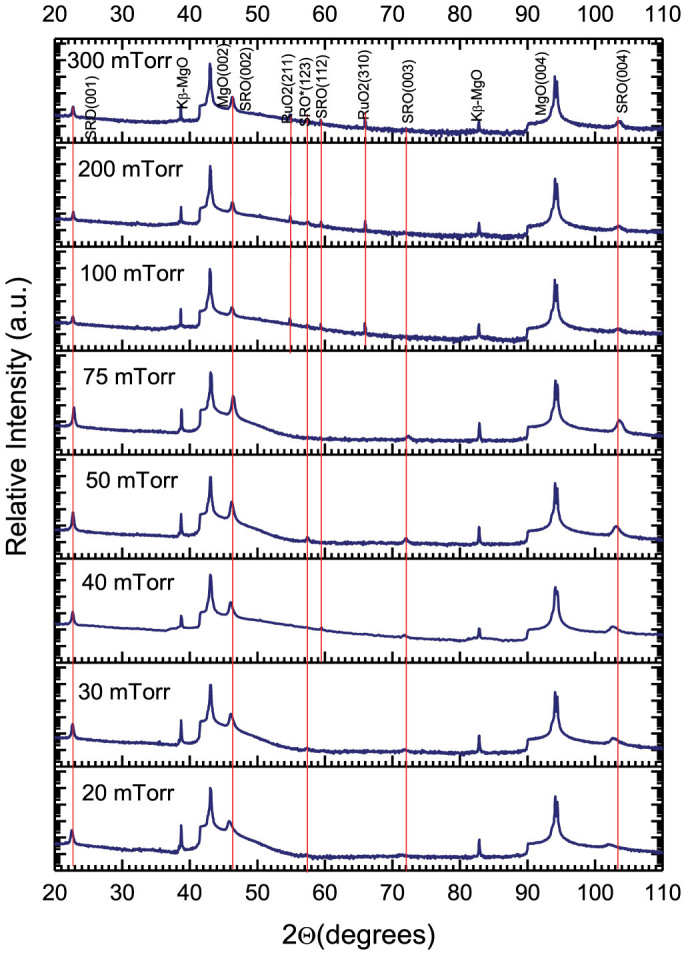
XRD patterns of SRO films grown on MgO (001) substrates at different oxygen pressures.

Figure 1 shows the XRD patterns for the samples grown at different oxygen pressures. All films were polycrystalline and exhibited a predominant (00l) orientation. In the samples deposited at high oxygen partial pressures (100–300 mTorr) inclusions of (112) and (123) oriented orthorhombic phase as well as RuO_2_ were observed. No other inclusions were observed in the film deposited at 75 mTorr. Below this pressure, growth along the (123) direction can be observed. All the films exhibited a smooth surface, with a roughness of 5–10 nm, as measured by contact-mode AFM, making them suitable for optical applications.

**Figure 2:**
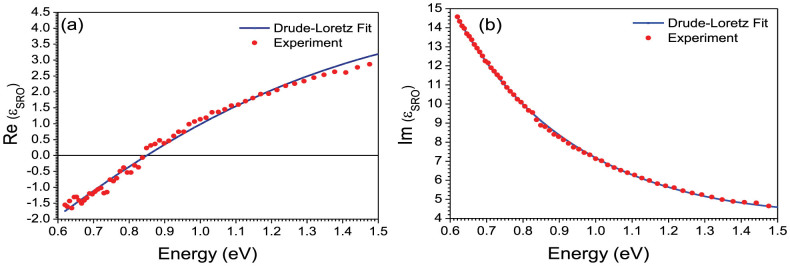
Experimental and fitted values of the (a) real and (b) imaginary parts of the dielectric constant of the 100 nm thick SrRuO_3_ film on MgO substrate grown at 100 mTorr oxygen pressure.

Figure 2 shows, as an example, the experimentally determined optical constants for the film prepared at 100 mTorr O_2_ pressure. The real part of the dielectric constant monotonously increases, while the imaginary part decreases in this spectral range. In order to understand the nature of the optical properties, the spectral dependence of the permittivity was fitted using the Drude model with a Lorentz-type oscillator to account for the interband transition in ruthenate materials^20^:
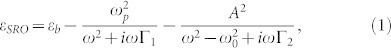
where *ε*_b_ is the background permittivity, ω_p_ is the plasma frequency, ω_0_ is the frequency of the interband transition, *A* is a constant, and Γ_1_ and Γ_2_ are the damping frequencies for the free and bound electrons, respectively. The best fit (Figure 2) was obtained for *ε*_b_ = 2.67, ω_p_ = 3.41 eV, *A* = 6.98 eV, ω_0_ = 3.38 eV, Γ_1_ = 0.98 eV and Γ_2_ = 3.23 eV with the goodness of fit test χ^2^ = 1.33. The fitting parameters were re-evaluated for all the other films and are in good agreement with optical measurements (similar to Figure 2, not shown) and the above parameters. The contribution of the additional RuO_2_ phases to the results in the examined wavelength range (0.6–1.5 eV) was negligible. The obtained spectral dependencies show that in the near infrared (NIR) regime (<1.4 eV), the Drude part of the permittivity dominates, describing well the spectral behavior of both the real and imaginary part of the SRO film dielectric constant (Figure 2). As wavelength increases, the ENZ frequency is reached at 0.84 eV (1470 nm), so that for the photon energies below the ENZ, Re(*ε_SRO_*) becomes negative in the measured spectral range up to 2000 nm. Conversely, for the same wavelength range, losses (i.e., Im(*ε_SRO_*)) increase monotonously. In the UV-VIS spectral range (>1.4 eV), a non-monotonous behavior is observed which is related to the interband transition resonance, in good agreement with Lee et al.^19^ Around the ENZ wavelength, losses of SRO are higher than Indium Tin Oxide (ITO) thin films^23^, however, SRO exhibits better temperature stability and minimal surface roughness (few nm) compared to ITO and its hybrids (typically tens of nm), making it more suitable for high-temperature and perfect for absorber applications.

## Discussion

**Figure 3:**
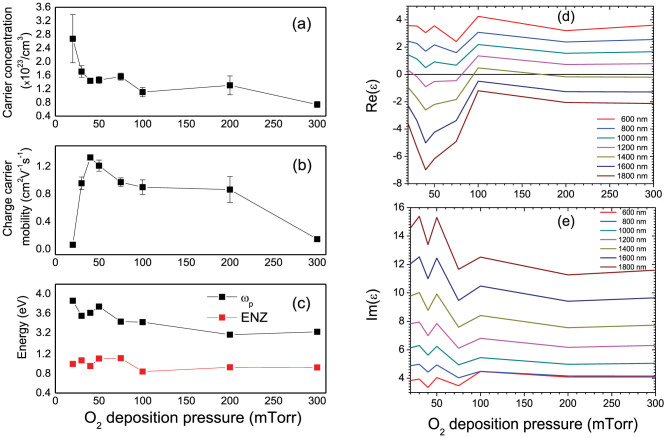
The dependence on oxygen pressure of (a) carrier concentration n, (b) carrier mobility μ, (c) plasma frequency ω_p_ and ENZ frequency, (d) real and (e) imaginary parts of the dielectric constant of the 100 nm thick SrRuO_3_ films, measured at various wavelengths from 600 nm (VIS) to 1800 nm (IR).

All the studied films exhibit n-type conductivity and ohmic transfer function. The electron concentration (Figure 3a) decreases with the increasing pressure, hinting at the influence of trapped charges, produced by defects in the thin films or at the film/substrate interface. At the same time, the electron mobility (Figure 3b) is the largest for the deposition pressures about 40–50 mTorr, while drops sharply for low O_2_ pressures and slowly decreases for larger ones. This observation manifests that the electron mobility in the SRO films is mainly influenced by the oxygen vacancies which are usually present in the films deposited at low O_2_ pressure as well as the scattering on grains boundaries with different orientation which were observed in the films deposited at high (100–300 mTorr) O_2_ pressure.

The plasma frequency extracted from the fitted experimental data (Figure 3c) decreases with the pressure following the trend of the measured electron concentration. The slight fluctuations of the plasma frequency are probably related to the free-charge trapping on the defect states modified in different growth conditions. It is interesting to note that the charge carrier concentration in the studied SRO films is on average approximately 10 times higher than that of Au or Ag, while the carrier mobility is about 100 times lower. The highest charge carrier concentration at low O_2_ deposition pressure is related to the contribution of the non-equilibrium charge carriers generated due to the non-stoichiometric charged defects (e.g., Oxygen vacancies) in the films. Their concentration reduces with the increase of the O_2_ deposition pressure and improvement of the SRO film crystal structure. This is further confirmed by the measured extremely low carrier mobility in films grown at O_2_ deposition pressure below 50 mTorr, as the non-equilibrium charge carriers are usually trapped by the defects^24^. The carrier concentrations measured for the films grown at O_2_ deposition pressure above 50 mTorr are in agreement with the previously observed values^25^,^26^.

Comparing the electron concentrations and plasma frequencies of the SRO and plasmonic metals, one can notice the increased effective electron mass in SRO as should be expected^27^,^28^. The cross-over (ENZ regime) from dielectric (positive permittivity) to plasmonic (negative permittivity) behavior takes place in the spectral range 1.11–1.47 eV, depending on the oxygen pressure (Figure 3c). For all the studied films, plasmonic behavior is observed in the infrared spectral range. In this spectral range, the absorption (Im(ε)) is comparable to that of Au for the films deposited at higher than 75 mTorr oxygen pressures.

The optical response, measured at various wavelengths from 600 nm (VIS) to 1800 nm (IR), corresponds well to the electric properties (Figure 3d) with the decreased Re(ε) for low pressures below 100 mTorr corresponding to the increased carrier concentration and, thus, stronger free-electron contribution in Equation (1). The decrease in mobility (and thus, increased scattering) can explain the increasing Re(ε) for pressures lower than 40 mTorr. (Please note that DC and optical-frequency scattering loss may not necessarily be the same). Nevertheless, the electron concentration is high enough to provide negative permittivity at infrared wavelengths for all deposition parameters. The optical losses (the imaginary part of the dielectric constant), have decreasing trend on the deposition pressure (Figure 3e), again in agreement with the electron concentration and the Drude model (Equaton 1). This behavior is increasingly pronounced for longer wavelengths where the free-electron (Drude) part of permittivity becomes dominant.

Other conductive oxides with the ENZ frequency in the telecommunication range, such as highly-doped semiconductors ZnO: Al, Ga or ITO may provide lower loss compared to SRO, and have good figure of merit for transformation optics applications (which is inversely proportional to loss)^3^. SRO benefits from the increased loss while maintaining the ENZ properties in the applications where the balance between the field penetration and dissipation is important, such as metamaterial-based absorbers, heat-generation in the near-IR and plasmonic hot-electron sources^26^.

In conclusion, thin SRO films of optical quality were grown on MgO (001) by pulsed laser deposition. The influence of the oxygen pressure (20–300 mTorr) on structure, charge carrier dynamics and optical properties were investigated. The best crystalline structure, characterized by the absence of extra phases or directions of growth, was obtained at the oxygen deposition pressure of 75 mTorr. The investigated films exhibit plasmonic behavior in the near-infrared spectral range with the plasma frequency at 3.16–3.86 eV and epsilon-near-zero behavior at 1.11–1.47 μm, depending on the deposition conditions. Oxygen pressure of 100–200 mTorr provides best electronic and optical properties, in this range of deposition parameters, for plasmonic applications of the films. The obtained parameters show the suitability of SrRuO_3_ thin films for plasmonic and metamaterial design applications which may range from the heat generating nanostructures in the near-infrared spectral range to metamaterial-based ideal absorbers and epsilon-near-zero components, which is difficult to achieve in the near-infrared with conventional plasmonic metals. In all these applications, the interplay between real and imaginary parts of the permittivity in a given spectral range is needed for optimization of the performance. This material is also compatible with and used in standard semiconductor technology and provides high temperature stability to up to 1200 K needed in many plasmonic applications, such as heat-assisted magnetic recording^29^.

## Methods

SrRuO_3_ thin films were deposited by PLD (Neocera Pioneer 150 system) from a sintered SrRuO_3_ target on (001)-oriented single crystal MgO substrates (Crystal GmbH, Berlin). The thin film deposition process was optimized to manufacture films with the best crystalline structure and electrical conductivity^30^. Therefore during the deposition the substrate temperature was kept at 700°C, while the oxygen pressure was varied between 20 and 300 mTorr. Detailed description of the thin film deposition process can be found elsewhere^30^. All films were analyzed ex-situ in open atmosphere, at room temperature.

A X-ray diffraction system (Brucker D2 Phaser), equipped with a graphite monochromator coupled with a scintillation counter detector using Cu K_α_ radiation λ = 1.5405 nm) was used in the Bragg-Brentano mode in order to investigate the crystallographic structure and phase composition of the films. Atomic force microscopy (AFM) was performed on a Brucker INNOVA instrument. The film thickness of all samples measured using a Dektak 150 surface profiler was 100 ± 5 nm. The electrical resistivity and charge carrier concentration and mobility of the films were investigated by Ecopia HMS-3000 Hall measurement system with Van der Pauw geometry at 0.55 T magnetic field, using soldered indium contacts.

The optical characterisation of the films was done using a Horiba Jobin-Yvon Uvisel 2 ellipsometer. Phase-modulation reflection ellipsometry was used for all measurements at a 70° angle of incidence. For all measurements, the signal was acquired from an elliptical spot size (2 mm × 0.7 mm). After the acquisition, the data were post-processed to determine optical parameters of the films in the wavelengths range 300–2000 nm. The MgO substrate optical constants^31^ and thickness of SrRuO_3_ were considered known and only the refractive index was fitted via the Marquardt minimization algorithm^32^. No pre-defined function (Drude, Tauc-Lorentz etc.) was used for the determination of the optical constants of SrRuO_3_; they were all directly fitted to the experimental data via the ‘point-by-point’ method.

## Author Contributions

P.K.P. and A.V.Z. conceived and designed the research. L.B., N.V. and B.Z. carried out the experiments. N.M.A. and S.A.M. contributed to the data analysis. All authors contributed to the manuscript writing and agreed on its final contents.
